# Correction: Novel insights into serodiagnosis and epidemiology of *Erysipelothrix rhusiopathiae*, a newly recognized pathogen in muskoxen (*Ovibos moschatus*)

**DOI:** 10.1371/journal.pone.0240760

**Published:** 2020-10-08

**Authors:** Fabien Mavrot, Karin Orsel, Wendy Hutchins, Layne G. Adams, Kimberlee Beckmen, John E. Blake, Sylvia L. Checkley, Tracy Davison, Juliette Di Francesco, Brett Elkin, Lisa-Marie Leclerc, Angela Schneider, Matilde Tomaselli, Susan J. Kutz

There are errors in the author affiliations. The correct affiliations are as follows:

Fabien Mavrot^1^, Karin Orsel^1^, Wendy Hutchins^2^, Layne G. Adams^3^, Kimberlee Beckmen^4^, John E. Blake^5^, Sylvia L. Checkley^1^, Tracy Davison^6^, Juliette Di Francesco^1^, Brett Elkin^6^, Lisa-Marie Leclerc^7^, Angela Schneider^1^, Matilde Tomaselli^1,8^, Susan J. Kutz^1^

**1** Faculty of Veterinary Medicine, University of Calgary, Calgary, Alberta, Canada **2** Cumming School of Medicine, University of Calgary, Calgary, Alberta, Canada **3** US Geological Survey, Alaska Science Center, Anchorage, Alaska, USA **4** Alaska Department of Fish and Game, Fairbanks, Alaska, USA **5** University of Fairbanks, Fairbanks, Alaska, USA **6** Government of the Northwest Territories, Yellowknife, Northwest Territories, Canada **7** Government of Nunavut, Kugluktuk, Nunavut, Canada **8** Canadian High Arctic Research Station, Polar Knowledge Canada, Cambridge Bay, Nunavut, Canada
