# Supplementary material for: Novel insights into serodiagnosis and epidemiology of Erysipelothrix rhusiopathiae, a newly recognized pathogen in muskoxen (Ovibos moschatus)
Source: PLoS One. 2020 Apr 21;15(4):e0231724. doi: 10.1371/journal.pone.0231724 (PMC7173868; doi:10.1371/journal.pone.0231724)
Supplement: Attachment — Submitted filename: Response to Reviewers_Mavrot_et_al.docx [file pone.0231724.s004.docx]

Please find below our responses to the comments of the academic editor and the reviewers (marked in bold).

Academic Editor comments:

**1.Please ensure that your manuscript meets PLOS ONE’s style requirements, including those for file naming.**

*We changed the file names to comply with the journal quidelines and adapted the text accordingly at lines 708-713*

**2. Thank you very much for including the following ethics statement on the submission details page:** **“The following wildlife sampling permits and ethic approvals were obtained for this study:
ADF&G ACUC Approved Protocols : 04-011, 06-08, 08-02, 2010-03, 2010-10R, 2011-012, 2012-04, 2013-18;
US Geological Survey Approved Protocol: 2009-01
Department of Environment of the Government of Nunavut: 2013-035, 2014-053, 2015-068 and 2016-058
Department of Natural Resources of the Government of the Northwest Territories: WL002097, WL002112, WL002853, WL003091, WL500098, WL500158,
WL500257,WL005627, WL005761, WL500018, WL 5004469
University of Calgary Animal Care and Use Permit (AC13-0121).” Please also include this ethics statement in the Methods section and clarify which permits or approvals are obtained for which samples from which countries.**

We have added the information at line 120-130

**3. We note that Figure 1 in your submission contain [map/satellite] images which may be copyrighted. All PLOS content is published under the Creative Commons Attribution License (CC BY 4.0), which means that the manuscript, images, and Supporting Information files will be freely available online, and any third party is permitted to access, download, copy, distribute, and use these materials in any way, even commercially, with proper attribution. For these reasons, we cannot publish previously copyrighted maps or satellite images created using proprietary data, such as Google software (Google Maps, Street View, and Earth). For more information, see our copyright guidelines:** [**http://journals.plos.org/plosone/s/licenses-and-copyright**](http://journals.plos.org/plosone/s/licenses-and-copyright)**.
 
We require you to either (1) present written permission from the copyright holder to publish these figures specifically under the CC BY 4.0 license, or (2) remove the figures from your submission:
 
a) You may seek permission from the original copyright holder of Figure 1 to publish the content specifically under the CC BY 4.0 license.  
 
We recommend that you contact the original copyright holder with the Content Permission Form (**[**http://journals.plos.org/plosone/s/file?id=7c09/content-permission-form.pdf**](http://journals.plos.org/plosone/s/file?id=7c09/content-permission-form.pdf)**) and the following text:
“I request permission for the open-access journal PLOS ONE to publish XXX under the Creative Commons Attribution License (CCAL) CC BY 4.0 (**[**http://creativecommons.org/licenses/by/4.0/**](http://creativecommons.org/licenses/by/4.0/)**). Please be aware that this license allows unrestricted use and distribution, even commercially, by third parties. Please reply and provide explicit written permission to publish XXX under a CC BY license and complete the attached form.”
 
Please upload the completed Content Permission Form or other proof of granted permissions as an "Other" file with your submission.
 
In the figure caption of the copyrighted figure, please include the following text: “Reprinted from [ref] under a CC BY license, with permission from [name of publisher], original copyright [original copyright year].”
 
b) If you are unable to obtain permission from the original copyright holder to publish these figures under the CC BY 4.0 license or if the copyright holder’s requirements are incompatible with the CC BY 4.0 license, please either i) remove the figure or ii) supply a replacement figure that complies with the CC BY 4.0 license. Please check copyright information on all replacement figures and update the figure caption with source information. If applicable, please specify in the figure caption text when a figure is similar but not identical to the original image and is therefore for illustrative purposes only.
The following resources for replacing copyrighted map figures may be helpful:
 
USGS National Map Viewer (public domain):** [**http://viewer.nationalmap.gov/viewer/**](http://viewer.nationalmap.gov/viewer/) **The Gateway to Astronaut Photography of Earth (public domain):** [**http://eol.jsc.nasa.gov/sseop/clickmap/**](http://eol.jsc.nasa.gov/sseop/clickmap/) **Maps at the CIA (public domain):** [**https://www.cia.gov/library/publications/the-world-factbook/index.html**](https://www.cia.gov/library/publications/the-world-factbook/index.html) **and** [**https://www.cia.gov/library/publications/cia-maps-publications/index.html**](https://www.cia.gov/library/publications/cia-maps-publications/index.html) **NASA Earth Observatory (public domain):** [**http://earthobservatory.nasa.gov/**](http://earthobservatory.nasa.gov/) **Landsat:** [**http://landsat.visibleearth.nasa.gov/**](http://landsat.visibleearth.nasa.gov/) **USGS EROS (Earth Resources Observatory and Science (EROS) Center) (public domain):** [**http://eros.usgs.gov/#**](http://eros.usgs.gov/) **Natural Earth (public domain):** [**http://www.naturalearthdata.com/**](http://www.naturalearthdata.com/)

Figure 1 is not a copy of an existing map and was created for the purpose of this publication. The GIS shapefile used to represent the sampling regions was done by the first author with the software QGIS using the free-hand polygon layer creation tool. The shapefiles used to represent the borders of Alaska and northern Canada were obtained from the website naturalearthdata.com. We modified the caption of figure 1 at lines 135-136 Material and Method section at line 191-192 to clarify this.

To determine each region, we used the exact locations of the sampled animals (not shown in the publication) and for Alaska, we additionally used the Game Management Units map described (among others) in Miller et al. 2017. For clarity purpose, we added a description on how regions were made at line 111-113 and added the relevant reference from Miller et al., 2017.

Reviewer 1:

**I would suggest to add in your conclusions that your approach could be used in other wildlife species as well in which a large number of samples is available, and there is an etiological agent recognized to affect the specific population**

We adapted the text as requested at lines 353-357

Reviewer 2:

**1. The authors’ conclusion and interpretation of data is based on that like in pigs, humoral immune responses in muskoxen are well induced by infection of E. rhusiopathiae. However, with experimental infections in cattle, I observed that E. rhusiopathiae does not induce strong humoral immune responses and the immunity does not last long. It may be possible in muskoxen as well. The authors should take it into consideration and can discuss this.**

We added some discussion points on those topics at lines 265-267 :

“There was a large difference between the means of the distributions of negative and positive samples in our dataset which allowed for a clear determination of cut-offs and indicate that muskoxen can display a strong immune response to the pathogen (the maximum PP in our set of sample was over 20 times the median value of all tested samples).”

and at lines 361-364:

“It must however be noted that the data presented here only reflects apparent seroprevalence in the investigated populations and is limited by many factors such as the lack of knowledge on antibody persistence, small sample sizes, bias due to opportunistic sampling, or missing information on the age and sex of exposed animals.”

**2. Do the authors have any information regarding the animals’ habitat environments? Were all the animals completely in the wild or could they get close to humans, or some farmed? This information is important, if the authors have that, please discuss in detail.**

We modified the text at line 120 to clarify that the animals are free-ranging and living in remote areas with no contact with domestic animals.

**3. L55: “Cl” should be spelled out on first appearance.**

Corrected as requested

**4. SpaA is not a membrane protein. So, “membrane proteins” should read “cell-wall-associated proteins” or “cell surface-located proteins” or “cell surface proteins”.**

Changed membrane protein to cell surface protein (line 144)

**5. Please check whether SpaA415 was coated on ELISA plate in PBS? Not in alkaline buffer?**

Indeed, we used a PBS buffer. The PBS recipe we use gives a final pH of 7.4 which is deemed sufficient for efficiently binding the antigen to Maxisorp plates. This part of the protocol follows exactly the instructions described in Gimenez-Lirola (2012) from which we adapted our ELISA. The ELISA protocol was modified in our study only to extend the range of species that can be tested, we saw no reason to change the buffer used for coating the plates.

**6. Page 14 Discussion L1 and L3: E. rhusiopathiae is not a syndrome or disease. Please correct them.**

We modified the text at line 250-252.

“In 2009-2013, *E. rhusiopathiae* was for the first time discovered and associated with high mortality rates in muskoxen in the Canadian Arctic Archipelago. Our first step was to assess if the bacterium was new to the Arctic or had historically been present.”

We also modified the text at line 89-92 to avoid the incorrect use of the term “disease syndrome”

“More recently, *E. rhusiopathiae* has been reported for the first time as a mortality cause in muskoxen between 2010-2013 [27], and has subsequentially been considered as a potential public health concern in the area [28]”

.

**7. Page 16, second paragraph can be “On Banks Island, several muskox die-offs were attributed to Yersinia pseudotuberculosis in the 1980’s and 1990’s, but in some cases, despite intensive investigation of Y. pseudotuberculosis at the time, macroscopic lesions were not consistent with yersiniosis (pulmonary edema rather than intestinal lesions [46]) and/or Y. pseudotuberculosis was not isolated from carcasses (1996 die-off [47]). As E. rhusiopathiae was, at that time unknown to affect muskoxen and not specifically looked for, this raises the question of whether those cases were, in fact, unrecognized E. rhusiopathiae infections. On Banks Island, seroprevalence alternated between years with higher values (1991, 2001, 2012) and years with lower values (1992, 2007), and the high seroprevalence documented in 1991 was not associated with any visible population decline or mortality events. The reason for the disassociation is unclear. However, this periodic increase in seroprevalence may suggest a cyclical pattern of outbreaks of E. rhusiopathiae similar to those described in other pathogens of free-ranging species [48,49]."**

We are grateful to reviewer#2 for bringing this part of the text to our attention. After reflecting on the changes proposed by reviewer#2 and discussing it with one of our co-authors (J. Blake) who was involved in the die-off investigation on Banks Island in the 1980s and 1990s, we have decided to remove the first half of the paragraph. (line 287-294).

“On Banks Island, seroprevalence alternated between years with higher values (1991, 2001, 2012) and years with lower values (1992, 2007). The small sample size and the gaps in the time series warrant a cautious interpretation of those results. However, this periodic increase in seroprevalence may suggest a cyclical pattern of outbreaks of *E. rhusiopathiae* similar to those described in other pathogens of free-ranging species [51,52]. “

**8. Figure 3: Prevalence (%)**

Corrected as requested

**9. Figure 3: Please add Population trend data for Victoria Island. If there is no data, it should be clarified in the text.**

Indeed, no reliable trend data are available for Victoria Island. We clarified this at line 227-229.

**Additional changes:**

During the revision process, there was an additional internal review required by our USGS colleagues. This, and our final review of the manuscript, has led to some additional minor edits for clarity. None of these edits have changed the findings or interpretations of the manuscript. These include:

Changed ‘strain’ to genotype for consistency throughout the text.

Line 65: changed “human population in the Arctic” to “wildlife-dependent human populations in the Arctic”

Line 130: added: “Details on sample collected and their serostatus are given in the supplementary material.”

Lines 165 and 167: replaced “determine” with “estimate”

Line 221: added “when comparing seroprevalence estimates for 2000-2014 to 1984-1992” to clarify which trend we are referring to.

Line 222-224 : replaced: ”In GMU 22 and 26, the highest seroprevalence corresponded to the muskox population peak just before they started to decline and, in GMU 22, was concomitant with unusual mortalities and the detection of *E. rhusiopathiae* in muskox carcasses [1,24]” with ” In GMU 22 and 26, the highest seroprevalence recorded corresponded to periods just before or during population declines. Additionally, in GMU 22, the peak seroprevalence was concomitant with unusual mortalities and the detection of *E. rhusiopathiae* in muskox carcasses [1,24]”.

Line 244: In the revision we realized that the phrase ‘’ the sampling period of 2011-2017 from 4.3 to 25%’ was a carry over from a much earlier draft of the manuscript and previous analysis where multiple locations had been combined. We had subsequently organized the data in more refined geographic units which made much more biological sense and this is how all the data have been presented throughout, with the exception of this one oversight. The phrase has now been replaced with “2011-2015 from 4.3 to 41.7%”. This correction does not affect the results nor interpretation of the data.

Line 274: Changed “established” to “estimated” and removed “effectively”. This was done for clarity.

Line 275: added ”as either positive or negative“.

Line 282: Replaced “and the increase in seroprevalence on these islands documented in this work” with “and the increase in seroprevalence documented on Victoria Island subsequent to the mortalities”. This was done as the data for Banks Island is not sufficient to make the statement for both islands.

Line 304-306: Replaced ”However, in two regions (GMU 22 and 26), the increase in seroprevalence since 2000, with the highest seroprevalence occurring concurrently with a peak in the muskox population, suggests that the epidemiology of the pathogen may have changed.” with “However, in two regions (GMU 22 and 26), yearly seroprevalences over 50% documented after 2000 indicate a possible increase in the presence of the pathogen in these regions.” This was done to simplify and clarify the text.

Line 312-313: Replaced: “has increased in some North American muskox populations in recent years” with “has increased in some North American muskox populations coincident with observed mortality events and population level declines”

Line 316: To improve clarity, we shortened the section “In this latter case, other mechanisms such as host density-dependent pathogen abundance [53] may play a role in the documented increase in *E. rhusiopathiae* occurrence. Furthermore, stress has been implicated in facilitating infections with *E. rhusiopathiae* in multiple species [54–56] and could be a contributing factor for muskoxen as well.” with “In this latter case, mechanisms such as host density-dependent pathogen abundance [53] and stress[54–56] may facilitate negative outcomes of infections with *E. rhusiopathiae* [54–56] and could be a contributing factor for muskoxen as well.”

Line 329-332: Added “Additionally, in our dataset, high seroprevalence against *E. rhusiopathiae* did not always fit known mortality events or population declines. This can be explained by low observation pressure and underreporting of mortalities [19] but also by the fact that the drivers of muskox population dynamics are likely to be more complex.”

Line 331: Replaced “and, in all likelihood, the drivers of muskox population dynamics are more complex.” with “but also by the fact that the drivers of muskox population dynamics are likely to be more complex.”

Line 339: removed “wide”

Line 346: removed “at least”

Line 350: To improve clarity, we replaced “…to establish the widespread historical exposure to a previously unknown Arctic pathogen in several North American populations” with “…to document the widespread historical exposure in several North American populations of a pathogen that was until recently not known to infect this species.”

Line 357: Replaced “the historical occurrence” with “historical seroprevalence”

Line 370: added: “documented for other animal species,” because, as of yet, there are no documented cases of transmission of *E. rhusiopathiae* from muskoxen to people.

References:

Giménez-Lirola, L. G., Xiao, C. T., Halbur, P. G. & Opriessnig, T. Development of a novel fluorescent microbead-based immunoassay and comparison with three enzyme-linked immunoassays for detection of anti-Erysipelothrix spp. IgG antibodies in pigs with known and unknown exposure. Journal of Microbiological Methods 91, 73–79 (2012).

Miller, S. D., Schoen, J. W. & Schwartz, C. C. Trends in brown bear reduction efforts in Alaska, 1980–2017. ursu 28, 135–149 (2017).
